# Decomposition techniques with mixed integer programming and heuristics for home healthcare planning

**DOI:** 10.1007/s10479-016-2352-8

**Published:** 2016-10-24

**Authors:** Wasakorn Laesanklang, Dario Landa-Silva

**Affiliations:** ASAP Research Group, School of Computer Science, The University of Nottingham, Jubilee Campus, Wollaton Road, Nottingham, NG8 1BB UK

**Keywords:** Home healthcare planning, Workforce scheduling and routing, Mixed integer programming, Problem decomposition, Heuristic decomposition

## Abstract

We tackle home healthcare planning scenarios in the UK using decomposition methods that incorporate mixed integer programming solvers and heuristics. Home healthcare planning is a difficult problem that integrates aspects from scheduling and routing. Solving real-world size instances of these problems still presents a significant challenge to modern exact optimization solvers. Nevertheless, we propose decomposition techniques to harness the power of such solvers while still offering a practical approach to produce high-quality solutions to real-world problem instances. We first decompose the problem into several smaller sub-problems. Next, mixed integer programming and/or heuristics are used to tackle the sub-problems. Finally, the sub-problem solutions are combined into a single valid solution for the whole problem. The different decomposition methods differ in the way in which sub-problems are generated and the way in which conflicting assignments are tackled (i.e. avoided or repaired). We present the results obtained by the proposed decomposition methods and compare them to solutions obtained with other methods. In addition, we conduct a study that reveals how the different steps in the proposed method contribute to those results. The main contribution of this paper is a better understanding of effective ways to combine mixed integer programming within effective decomposition methods to solve real-world instances of home healthcare planning problems in practical computation time.

## Introduction

Home healthcare planning (HHC) is a type of workforce scheduling and routing problem (Castillo-Salazar et al. 2014). In HHC the problem is to allocate nurses or care workers to deliver care services at the patient’s home. The problem involves producing a job schedule and a route for every worker while its solution satisfies business requirements such as workers qualifications and skills, task requirements, travelling requirements, etc. at the lowest operational cost. Section 2 describes the HHC problem and instances tackled in this paper.

The HHC problem can be formulated as an integer programming or as a mixed integer programming model. It has been shown that solving the problem with traditional optimization solvers is not computationally efficient (Borsani et al. 2006; Bredström and Rönnqvist 2007). Therefore, several solution approaches use heuristic methods (Brandimarte 1993; Akjiratikarl et al. 2007; Pillac et al. 2012; Pinheiro et al. 2015; Algethami and Landa-Silva 2015). However, there are ways to harness the power of mathematical programming solvers while still achieving good efficiency in terms of computational time. Here, we investigate problem decomposition techniques that perform well on a range of HHC real-world instances. Section 3 reviews the key literature on decomposition techniques to put our work into context.

A Geographical Decomposition with Conflict Avoidance (GDCA) method that splits the problem into sub-problems defined by geographical regions was presented in Laesanklang et al. (2015). This method keeps track of workers assigned in sub-problems in order to prevent conflicting assignments (assigning a worker to more than one task at the same time). Sub-problems are solved in sequence and several heuristics to establish such sequence are proposed. The main drawback of this GDCA method is that the solution quality depends heavily on the sub-problem solving sequence. Finding an effective sequencing rule that allows to always find a good complete solution is very difficult. Further details of this method are given in Sect. 4. Then, we propose here an improved decomposition technique, called Geographical Decomposition with Conflict Repair (GDCR), where the solving sequence is no longer required. This method allows conflicting assignments to happen which are then repaired later to produce a valid solution. Further details of this method are given in Sect. 5. Another decomposition method presented here is an iterative approach called Repeated Decomposition and Conflict Repair (RDCR) that combines decomposition and conflicting assignments repair iteratively, resulting in an improvement on the computational time. Further details of this method are given in Sect. 6. Section 7 presents a comparative study between solutions produced by GDCA, GDCR, RDCR, a baseline heuristic and a practitioner (expert human planner).

In summary, the main contribution of this paper is to present two improved decomposition techniques to tackle real-world instances of the home healthcare planning (HHC) problem in the UK. These techniques are the *Geographical Decomposition with Conflict Repair (GDCR)* and the *Repeated Decomposition and Conflict Repair (RDCR)*, which harness the power of modern mixed-integer programming solvers in order to produce high-quality solutions in practical computation time. This paper also conducts a study of the contribution that the various steps make to the performance of these proposed techniques and a comparative study against solutions produced by a baseline heuristic and the human planner. Section 8 of the paper gives a summary, conclusions and proposed future research.

## The home healthcare planning problem

The goal in home healthcare planning (HHC) is to assign to each worker, a set of tasks to be performed where each task is usually at a different geographical location (i.e. the patient’s home). A path is a series of tasks to be carried out by a worker within the planning period. A solution to a HHC problem instance is a collection of paths that cover the set of tasks. The solution should also satisfy other conditions such as task requirements, appointment times, required workers qualifications and skills, workers availability, restricted working regions, working time limits, etc. A good-quality solution should have low operational cost. This section describes the real-world HHC problem tackled here and the problem instances provided by our industrial partner. A mixed integer programming (MIP) formulation for this scenario is presented next.

### Formulation of constraints

The HHC problem tackled here can be represented by a graph $G=(V,E)$ where *V* is a set of nodes and *E* is a set of edges between nodes. The set of nodes $V= D \cup T \cup D'$ where *T* is a set of visiting nodes or tasks, *D* and $D'$ are sets of source and sink nodes respectively (e.g. the worker’s home). The set of edges *E* represents a set of links between two nodes (e.g. between two task locations or between the workers home and a task location). For convenience, we define $V^S=D\cup T$ as nodes that have leaving edges and and $V^N = D'\cup T$ as nodes that have incoming edges. The set of workers is represented by *K*. A path is the assignment of a set of edges from *E* to worker $k \in K$. For example, a path is as series of visits to task locations performed by a worker starting and ending at the worker’s home. A binary decision variable $x_{i,j}^k$ represents the assignment of edges to worker *k*, then $x_{i,j}^k=1$ if the edge between nodes *i* and *j* is assigned to worker *k* (i.e. worker *k* carries out task *i* followed by task *j*), otherwise $x_{i,j}^k=0$.

In real-world HHC scenarios like the ones considered here, it is possible that some tasks are left unassigned as there is not enough workforce or no worker has the required qualifications/skills. In such cases, an integer variable $y_j$ is used to indicate the number of unsatisfied assignments for task *j* (i.e. task may require more than one worker) (Bredström and Rönnqvist 2008; Rasmussen et al. 2012). If task *j* is fully assigned then $y_j=0$, otherwise $y_j$ takes a positive integer value equal to the number of workers required to the task. Constraint (1) ensures this requirement is met even for tasks that are unassigned, $r_j$ is the number of workers required for task *j*.

1 $$\begin{aligned} \sum _{k \in K} \sum _{i \in V^S} x_{i,j}^k + y_j = r_j, \quad \forall j \in T \end{aligned}$$

A path or sequence of tasks can be indicated by a set of variables $x_{i,j}^k = 1$ ensuring that they form a connected sequence of edges for each worker *k* as given by constraint (2).

2 $$\begin{aligned} \sum _{i \in V^S} x_{i,j}^k = \sum _{n \in V^N} x_{j,n}^k, \quad \forall j \in T, \forall k \in K \end{aligned}$$

In addition, the path for each worker *k* should begin at a start location and terminate at an end location (e.g. their home or a central office). The start location and the end location of worker *k* are $D_k$ and $D'_k$, respectively. The condition is enforced by constraints (3) and (4). These constraints only apply to the nodes representing the worker’s start and end locations. Workers may leave their start location and enter their end location at most once (although the start and end locations may be different) as expressed by constraints (5) and (6) respectively.

3 $$\begin{aligned} \sum _{j \in V^N} x_{n,j}^k\ge & {} \sum _{j \in V^N}x_{i,j}^k ,\quad \forall k \in K, \forall i \in T, \exists n \in D \end{aligned}$$

4 $$\begin{aligned} \sum _{i \in V^S} x_{i,n}^k\ge & {} \sum _{i \in V^S}x_{i,j}^k,\quad \forall k \in K, \forall j \in T, \exists n \in D' \end{aligned}$$

5 $$\begin{aligned} \sum _{j \in V^N} x_{i,j}^k\le & {} 1,\quad \forall i \in D, \forall k \in K \end{aligned}$$

6 $$\begin{aligned} \sum _{i \in V^S} x_{i,j}^k\le & {} 1,\quad \forall j \in D', \forall k \in K \end{aligned}$$

The problem also requires that workers have the required skills for every assigned task. Let a binary parameter $q^k_j$ represent a qualification parameter where $q^k_j = 1$ when a worker *k* has the skills to take visit *j*, and $q^k_j=0$ otherwise. Only qualified workers can make the visit as indicated by constraint (7).

7 $$\begin{aligned} x_{i,j}^k \le q^k_j,\quad \forall k \in K, \forall i \in V^S,\forall j \in T \end{aligned}$$

Travelling between task locations must be feasible in terms of travel time. Decision variable $a_j^k$ takes a positive fractional value that gives the arrival time of worker *k* to the location of task *j*. Note that the maximum arrival time value here is 1440 minutes, which is equivalent to the 24th hour of the day. Let $a_i^k$, $a_j^k$ be the arrival times of worker *k* at the locations of task *i* and task *j* respectively. The arrival time at task *j* must consider the time duration $\delta _i$ spent on performing task *i* and the travelling time $t_{i,j}$ between task node *i* and task node *j*. This is enforced by constraint (8) where *M* is a large constant number.

8 $$\begin{aligned} a_j^k + M(1-x_{i,j}^k) \ge a_i^k + x_{i,j}^k t_{i,j}+\delta _i ,\quad \forall k \in K, \forall i \in V^S, \forall j \in V^N \end{aligned}$$

A worker *k* must arrive at task node *j* within the given time window. For task *j*, the earliest arrival time is $w_j^L$ and the latest arrival time is $w_j^U$. This requirement is enforced by constraint (9).

9 $$\begin{aligned} w_j^L \le a_j^k \le w_j^U ,\quad \forall j \in T, \forall k \in K \end{aligned}$$

Time availability can be different for each worker according to their individual contracts. We adopt the availability constraint from the literature (Trautsamwieser and Hirsch 2011) which defines a time availability period for each worker. The shift start time and shift end time of the worker *k* are indicated by $\alpha _L^k$ and $\alpha _U^k$ respectively. However, in the scenarios tackled in this paper, tasks can be assigned outside the worker’s shift but subject to a penalty cost. In order to indicate this, we introduce a binary decision variable $\omega ^k_j=1$ to indicate such penalisation. The time availability constraints for worker *k* are given by expressions (10) and (11).

10 $$\begin{aligned} \alpha ^k_L - a^k_j \le M(1-x^k_{i,j}+\omega ^k_j), \quad \forall k \in K, \forall i \in D\cup T, \forall j \in T \end{aligned}$$

11 $$\begin{aligned} a^k_j + \delta _j -\alpha ^k_U \le M(1-x^k_{i,j}+\omega ^k_j),\quad \forall k \in K, \forall i \in D\cup T, \forall j \in T \end{aligned}$$

Another working regulation in the HHC scenarios tackled here is not to exceed the maximum working hours for each worker. Each task *j* requires $\delta _j$ minutes to be completed. The maximum working hours for worker *k* is given by $h^k$. Constraint constraint (12) enforces this regulation.

12 $$\begin{aligned} \sum _{i \in V^S} \sum _{j \in T} x_{i,j}^k \delta _j \le h^k,\quad \forall k \in K \end{aligned}$$

In our HHC scenarios, each worker is associated to a set of geographical regions defined by the service provider. In short, a geographical region contains several task locations and a task location may have several tasks to be assigned. Ideally, a worker should only be assigned to tasks in those geographical regions. However, if necessary, a worker can be asked to travel to locations outside their geographical regions subject to some penalty cost. We define a binary parameter $\gamma _j^k=1$ to indicate that task *j* is located in the worker’s regions and $\gamma _j^k=0$ otherwise. We define a binary variable $\psi ^k_j=1$ to indicate that task *j* assigned to worker *k* is outside the worker’s regions, and $\psi ^k_j=0$ otherwise. Constraint (13) presents the relation between these binary variables for the different possible cases.

13 $$\begin{aligned} \sum _{i \in V^S}x_{i,j}^k - \psi ^k_j \le \gamma _j^k,\quad \forall k \in K, \forall j \in T \end{aligned}$$

Note that some of the constraints expressed in the above MIP formulation actually express soft requirements in our HHC scenarios. These are constraint (1) (tasks may be left unassigned), constraints (10) and (11) (workers may be asked to work outside their shift hours) and constraint (13) (workers may be asked to work outside their geographical regions). Later for one of the methods described in this paper, constraints (10) and (11) are re-formulated to enforce the condition that workers must not work outside their shift hours.

### Formulation of the objective function

The objective function (14) to be minimized involves three costs: monetary cost, soft constraints penalty and preferences penalty. This objective function has been defined in consultation with our industrial partner as it seeks to incorporate the key aspects that make a high-quality solution: low operational cost and improved satisfaction of patients and workers. These costs are balanced into four priority levels, each corresponding to one of the weights $\lambda _1,{\ldots },\lambda _4$.

14 $$\begin{aligned}&\text {Min} ~~~ \lambda _1 \sum _{k \in K} \sum _{i \in V^S} \sum _{j \in V^N} \left( d_{i,j}+p^k_j \right) x_{i,j}^k +\,\lambda _2 \sum _{j\in T}\left( 3r_j - \sum _{i \in V^S}\sum _{k\in K}\rho ^k_j x^k_{i,j}\right) \nonumber \\&\quad \,+\,\lambda _3\sum _{k \in K}\sum _{j \in T}\left( \omega ^k_j+ \psi ^k_j\right) + \lambda _4 \sum _{j \in T} y_j \end{aligned}$$

The weights associated to each operational cost component should be set to values that clearly reflect the difference between the priority levels (Rasmussen et al. 2012; Castillo-Salazar et al. 2014). The highest priority is given to minimize unassigned tasks through weight $\lambda _4$. This is because the first priority of the service provider is to complete as many tasks as possible. The second highest priority is given to minimize the soft constraints penalty (i.e. number of worker time availability and working regions violations) through weight $\lambda _3$. This is because in practice the service provider may ask workers to undertake tasks that are outside their time availability and/or geographical region. The third priority is given to minimize the preferences penalty through weight $\lambda _2$. These preferences are expressed in our HHC scenarios and there are three types: preferred worker-client pairing, worker preferred region and client preferred skills. The degree of satisfaction of these preferences when assigning worker *k* to task *j* is given by $\rho ^k_j$ which has a value in the range [0, 3]. This is because the satisfaction of the three types of preferences for each assignment has a value in the range [0, 1] from not satisfied to satisfied. The satisfaction level is reverted to penalty by subtracting it from the full satisfaction score, which is $3r_j$ for each visit *j*. Finally, the fourth and lowest priority is given to minimize the monetary cost through weight $\lambda _1$. The monetary cost includes travelling cost $d_{i,j}$ and workers salary $p_j^k$ as calculated by the service provider in our HHC scenarios.

Note that in the above objective function, two of the four priority levels involve a cost related to the geographical regions. In the soft constraints penalty, assignments outside the worker’s available regions are penalized. In the preferences penalty, assignments made in a less-preferred region (but still within the worker’s available regions) are penalized. Note also that worker skills are involved in two parts of the model. The set of base worker skills required by each task is accounted in constraint (7). The client preferred skills accounted in the preferences penalty cost refers to additional skills that are desirable depending on the client to be served. With this weighted objective function based on priority levels, the total penalty due to violating all soft constraints is always higher than the total penalty due to violating all preferences, as this reflects practice in our HHC scenarios.

### Real-world problem instances

The problem instances used here were provided by our industrial partner. Their main business is to provide workforce management software as a service to a large number of home healthcare service providers. From their large number of real-world scenarios, they kindly provided the data for 6 different scenarios and 7 different planning periods resulting in 42 problem instances. We classified these instances in two groups: small and large. The small instances are those labelled WSRP-A-(01-07) and WSRP-B-(01-07). The large instances are those labelled WSRP-D-(01-07), WSRP-E-(01-07) and WSRP-F-(01-07). Table 1 shows the main features of these 42 problem instances. For each instance, the table shows: number of workers (|*K*|), number of different task locations (|*L*|), number of tasks to assign (|*T*|) and number of geographical regions (|*A*|). Problem instances in the group WSRP-C are different from the others in that they have a much larger workforce size (|*K*|). In these instances there are many tasks but in a relatively small number of locations. For example, problem instance WSRP-C-01 has $T=177$ tasks distributed in only $L=8$ locations. Note also that in these WSRP-C instances each geographical region includes only one task location, i.e. $A=L$. Therefore, these WSRP-C instances do not involve routing within a region but they may involve routing between regions.

**Table 1:** The HHC problem instances obtained from real-world operational scenarios

Set	|*K*|	|*L*|	|*T*|	|*A*|	Set	|*K*|	|*L*|	|*T*|	|*A*|
WSRP-A-01	23	25	31	6	WSRP-B-01	25	27	36	6
WSRP-A-02	22	24	31	4	WSRP-B-02	25	11	12	4
WSRP-A-03	22	28	38	5	WSRP-B-03	34	43	69	6
WSRP-A-04	19	22	28	3	WSRP-B-04	34	14	30	4
WSRP-A-05	19	9	13	3	WSRP-B-05	32	38	61	8
WSRP-A-06	21	22	28	7	WSRP-B-06	32	38	57	7
WSRP-A-07	21	9	13	3	WSRP-B-07	32	38	61	7
WSRP-C-01	1037	8	177	8	WSRP-D-01	164	233	483	13
WSRP-C-02	618	4	7	4	WSRP-D-02	166	215	454	12
WSRP-C-03	1077	7	150	7	WSRP-D-03	174	279	585	15
WSRP-C-04	979	8	32	8	WSRP-D-04	174	237	520	15
WSRP-C-05	821	6	29	6	WSRP-D-05	173	259	538	15
WSRP-C-06	816	11	158	11	WSRP-D-06	174	291	610	15
WSRP-C-07	349	5	6	6	WSRP-D-07	173	293	611	15
WSRP-E-01	243	239	418	13	WSRP-F-01	805	477	1211	45
WSRP-E-02	244	257	425	14	WSRP-F-02	769	496	1243	46
WSRP-E-03	267	264	462	15	WSRP-F-03	898	582	1479	54
WSRP-E-04	266	174	351	13	WSRP-F-04	789	513	1448	47
WSRP-E-05	278	263	461	15	WSRP-F-05	883	626	1599	59
WSRP-E-06	278	138	301	13	WSRP-F-06	783	565	1582	44
WSRP-E-07	302	276	498	16	WSRP-F-07	1011	711	1726	64

|*K*| number of workers, |*L*| number of task locations, |*T*| number of tasks, |*A*| number of geographical regions

As part of the project in which this research has been conducted, we developed a framework to facilitate the collaboration between researchers and practitioners (Pinheiro and Landa-Silva 2014). Among other things, this framework is used to process instances data and to validate/evaluate solutions according to the objective function (14). This framework has also facilitated the development and consistent evaluation of other solution techniques being investigated. The real-world instances and related documentation are available at the following location: https://drive.google.com/open?id=0B2OtHr1VocuSNGVOT2VSYmp6a2M. An analysis of these benchmark problem instances and a comparison of methodologies to solve them are presented in Pinheiro et al. (2016).

## Literature review

Although mathematical programming is effective for modelling real-world HHC scenarios like the one described above, solving large instances using MIP solvers is not yet very efficient in terms of computational time. Despite the fact that heuristic algorithms can be developed, here we are interested in investigating ways to harness the power of modern exact optimization solvers to produce high-quality solutions in practical computation time. One possibility is to use problem decomposition and next we review some of the literature relevant to our research.

### Traditional decomposition methods

Decomposition is a technique for tackling a large scale problem which cannot be handled with MIP solvers, the technique seeks to exploit the problem structure (Ralphs and Galati 2010). Decomposition methods have been applied to many problems such as aircraft routing and crew scheduling problem (Cordeau et al. 2001; Salazar-González 2014), manpower allocation problem (Dohn et al. 2009), employee tour scheduling problem (Ni and Abeledo 2007) and home healthcare scheduling (Rasmussen et al. 2012).

The principle in traditional decomposition methods is to improve solution bounds (upper and/or lower bound) (Ralphs and Galati 2010). The same principle is applied in the general branch and bound algorithm where bounds are narrowed by computing the linear programming (LP) relaxation or other relaxation techniques that provide better bounds, for example, Lagrangian Relaxation (Fisher 2004). Traditional decomposition is usually beneficial when the optimization problem can be defined in some specific structure such as block-diagonal structure so that it can be approached by optimizing blocks independently. There are two main decomposition approaches for exploiting problem structure: constraint decomposition and variable decomposition (Vanderbeck and Wolsey 2010).

The constraint decomposition method creates a compact problem by inserting approximation planes or constraints to get a better approximation. The planes could generate either outer approximation (cutting plane methods) (Kelley 1960) or inner approximation (Dantzig-Wolfe method Vanderbeck 2000, Lagrangian method Ruszczyński 1989). The additional plane generates a cut which eliminates part of the feasible region that does not contain an integer solution. Improved methods use both inner and outer approximations to get better LP bounds (Vanderbeck 2000). Thus, the problem must be derived in both primal and dual formulations in order to apply a two-way approximation.

The variable decomposition method is applied to problems where decision variables can be separated mostly into two types (Benders 1962). The method solves the problem in two stages. The first stage chooses a set of integer variables and finds values for them. The second stage finds the optimal solution for the other variables subject to the values given to the first group of variables in the first stage. Benders’ decomposition is a method representing this type of approach. It has been applied to many problems such as network design (Costa 2005), scheduling and routing of automated guided vehicles (Corréa et al. 2007) and tour scheduling (Rekik et al. 2004).

### Heuristic decomposition methods

Heuristic decomposition methods basically seek for a feasible solution and are based on decomposing both variables and constraints. A difference with traditional decomposition methods is that heuristic decomposition discards bounds improvement. Therefore, the process is significantly faster because the repeated optimization process to close the gap between lower and upper bounds is removed.

In essence, heuristic decomposition methods reduce the problem size by partitioning the problem into smaller sub-problems. The partitioning can be done by using a general scheme such as splitting the whole problem into equally sized sub-problems. Also, the partitioning can be done based on some specific problem feature, an example is the steel plant production scheduling problem where the steel making process was used as a splitting rule (Harjunkoski and Grossmann 2001). The sub-problems created by the partitioning rule are usually defined in a mathematical model. Hence, solving the sub-problems can be approached with a mathematical programming solver. The sub-problem solving process can be done independently or hierarchically. An example of independent sub-problem solving is the time-based decomposition applied to a scheduling model where the time horizon was used as splitting criterion (Bassett et al. 1996). Hierarchical sub-problem solving requires a solving order for the sub-problems. A tier-based hierarchical decomposition defines each tier as a different model, for example batch plant design and planning (Subrahmanyam et al. 1996), a warehouse location-routing problem (Perl and Daskin 1985), multi-depot location routing problem (Wu et al. 2002).

Geographical decomposition is an approach that can be applied to partition problems where geographical regions are defined like in vehicle routing problems. Considering geographical proximity in the decomposition can help to generate efficient routing paths (Reimann et al. 2004). For example, polar coordinates partitioning can be applied to problems where there is a depot located on the centre and visiting locations are distributed around the depot (Taillard 1993). Geographical decomposition can employ clustering algorithms for the partitioning (Campbell and Savelsbergh 2004). However, clustering alone might not benefit the mathematical solver if the clusters are too large for the solver to tackle. Rules can also be applied to control the size of sub-problems, for example to limit the number of customers by merging customers within a small neighbourhood into one job. This approach was used in a multi-carrier transportation planning problem where delivery jobs are assigned to multiple carrier companies subjected to their operational cost (Landa-Silva et al. 2011).

The next section reviews Geographical Decomposition with Conflict Avoidance (GDCA). Furthermore, we propose two improved decomposition methods: Geographical Decomposition with Conflict Repair (GDCR), and Repeated Decomposition and Conflict Repair (RDCR) which will be described in Sects. 5 and 6 respectively.

## Geographical decomposition with conflict avoidance (GDCA)

This section describes the implementation of the Geographical Decomposition with Conflict Avoidance (GDCA), a heuristic decomposition technique designed to tackle the HHC problem (Laesanklang et al. 2015). Basically, it decomposes an instance into geographical regions as featured by the problem. The GDCA splits a main problem into smaller sub-problems each representing a different geographical region and small enough to be tackled with an MIP solver. We note that the solution produced by GDCA is not guaranteed to be optimal.

**Fig. 1:**
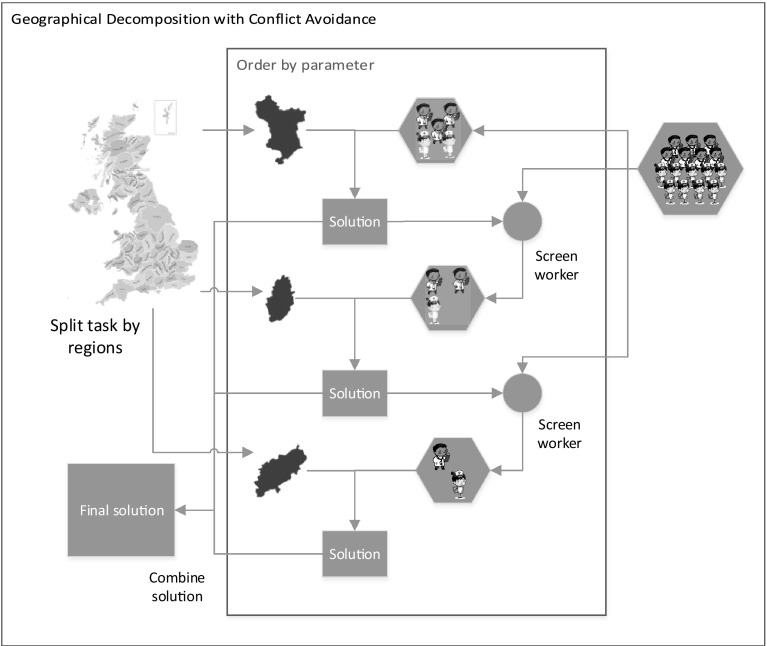
The Geographical Decomposition with Conflict Avoidance (GDCA) Approach

Figure 1 shows the outline of the GDCA. On the left, tasks are partitioned into geographical regions. On the right, workers are selected by their geographical region availability. Both components are combined into sub-problems as illustrated in the middle of the figure. Sub-problems are ordered based on some criterion and they are tackled with the MIP solver in that order. After a sub-problem is solved, the available workforce is updated so that the next sub-problem to be solved does not generate *conflicting assignments*, i.e. tasks overlapping in time assigned to the same worker. Thus, in this approach conflicts are avoided as sub-problems are solved in sequence. Finally, sub-problem solutions are combined into a final solution, i.e. a set of paths one for each worker.
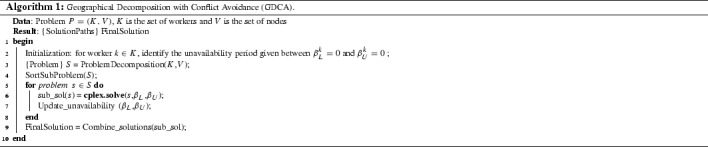


Algorithm 1 outlines the GDCA method which takes a problem instance and generates a solution. A problem instance *P* can be defined by a set of workers *K* and a set of nodes *V* (tasks and start/end locations). The algorithm starts by identifying the unavailability time period between $\beta ^k_L=0$ and $\beta ^k_U=0$ for every worker $k \in K$. Next, the problem instance *P* is split into sub-problems $s \in S$ (step 3). Each sub-problem *s* contains the task nodes located in the same geographical region and workers who are available to work in that region. Each sub-problem *s* also contains the start/end location nodes. Hence, each task is in exactly one sub-problem but a worker may be in more than one sub-problem. Each sub-problem is defined by the MIP model presented in Sect. 2 plus the additional constraints (15) and (16). The addition of these constraints means that the worker time availability constraint is enforced in GDCA and hence conflicting assignments are avoided. These constraints define the time period between $\beta ^k_L$ and $\beta ^k_U$ as unavailable. The binary variable $\zeta ^k$ is used to enforce the selection of only one side of the available time period, $\zeta ^k = 1$ for the time interval up to $\beta ^k_L$, and $\zeta ^k = 0$ for the time interval from $\beta ^k_U$ onwards. Sub-problems are then sorted by non-increasing number of tasks (step 4) and solved in that order (steps 5–8). For every solved sub-problem, $\beta ^k_L$ and $\beta ^k_U$ are updated for every worker $k \in K$.

15 $$\begin{aligned} a^k_j + \delta _j - \beta ^k_L \le M(2-x^k_{i,j}-\zeta ^k) \qquad \forall k \in K, \forall i \in V^S, \forall j \in V^N \end{aligned}$$

16 $$\begin{aligned} \beta ^k_U - a^k_j \le M(1-x^k_{i,j}+\zeta ^k) \qquad \forall k \in K, \forall i \in V^S, \forall j \in V^N \end{aligned}$$

After all sub-problems are solved, constructing the final solution requires combining the paths from all the sub-problems so that a worker has only one working path (step 9). Although each sub-problem provides a path per worker, a worker may be involved in multiple sub-problems hence resulting in multiple paths for that worker. Thus, these multiple paths are simply merged into one single path as follows. Let $\Phi ^k_1,\Phi ^k_2,\ldots ,\Phi ^k_n$ be the multiple paths for worker *k* given by solving *n* sub-problems. These paths have the same start location *d* and end location $d'$. Without loss of generality, assume $\Phi ^k_1$ is the earliest path, $\Phi ^k_2$ is the second earliest path and so on. The paths merging process takes the two earliest paths $\Phi ^k_1$ and $\Phi ^k_2$. Then, the ending edge of $\Phi ^k_1$ (connecting the last task *i* to the end node $d'$) and the starting edge of $\Phi ^k_2$ (connecting the start node *d* to the first task *j*) are removed. This is done by setting $x^k_{i,d^\prime } = 0$ and $x^k_{d,j}=0$. Next, the edge connecting *i* and *j* for worker *k* is selected by setting $x^k_{i,j} = 1$. Since *d* and $d^\prime$ is the same location thus

$$\begin{aligned} a^k_i + t_{i,d^\prime } \le a^k_{d^\prime } \le a^k_d < a^k_d + t_{d,j} \le a^k_j \end{aligned}$$

where $a^k_i, a^k_j, a^k_d, a^k_{d^\prime }$ are the arrival times at tasks *i* and *j*, start node *d* and end node $d^\prime$ respectively. Therefore, paths $\Phi ^k_1$ and $\Phi ^k_2$ are now merged. This process continues connecting the recently merged path to the next earliest path until a single path for worker *k* is formed.

As mentioned above, GDCA requires an ordering for solving the sub-problems. Our experiments did not identify an ordering criterion that performed the best on all instances. However, we observed that ordering the sub-problems by non-increasing order of the number of tasks provided good overall performance. Experimental results from using this setup on GDCA will be shown in Sect. 7 alongside with the results from other decomposition algorithms.

## Geographical decomposition with conflict repair (GDCR)

Solving the problem as a whole with an MIP solver is not practical for the case of instance sets WSRP-A, WSRP-B and some in WSRP-C, but the GDCA described above finds feasible solutions for all test instances. However, the solution quality obtained by GDCA still depends on the sub-problems solving sequence. Moreover, there seems to be no solving sequence that works better for all instances. Therefore, we propose a decomposition approach which does not require a solving sequence, Geographical Decomposition with Conflict Repair (GDCR). Here, sub-problems are tackled in no specific order. The method consists of three stages: geographical-based decomposition, conflicting assignments repair and heuristic assignment. The first two stages achieve most of the tasks assignments of a problem instance, but the heuristic assignment is crucial to complete the whole solution.

**Fig. 2:**
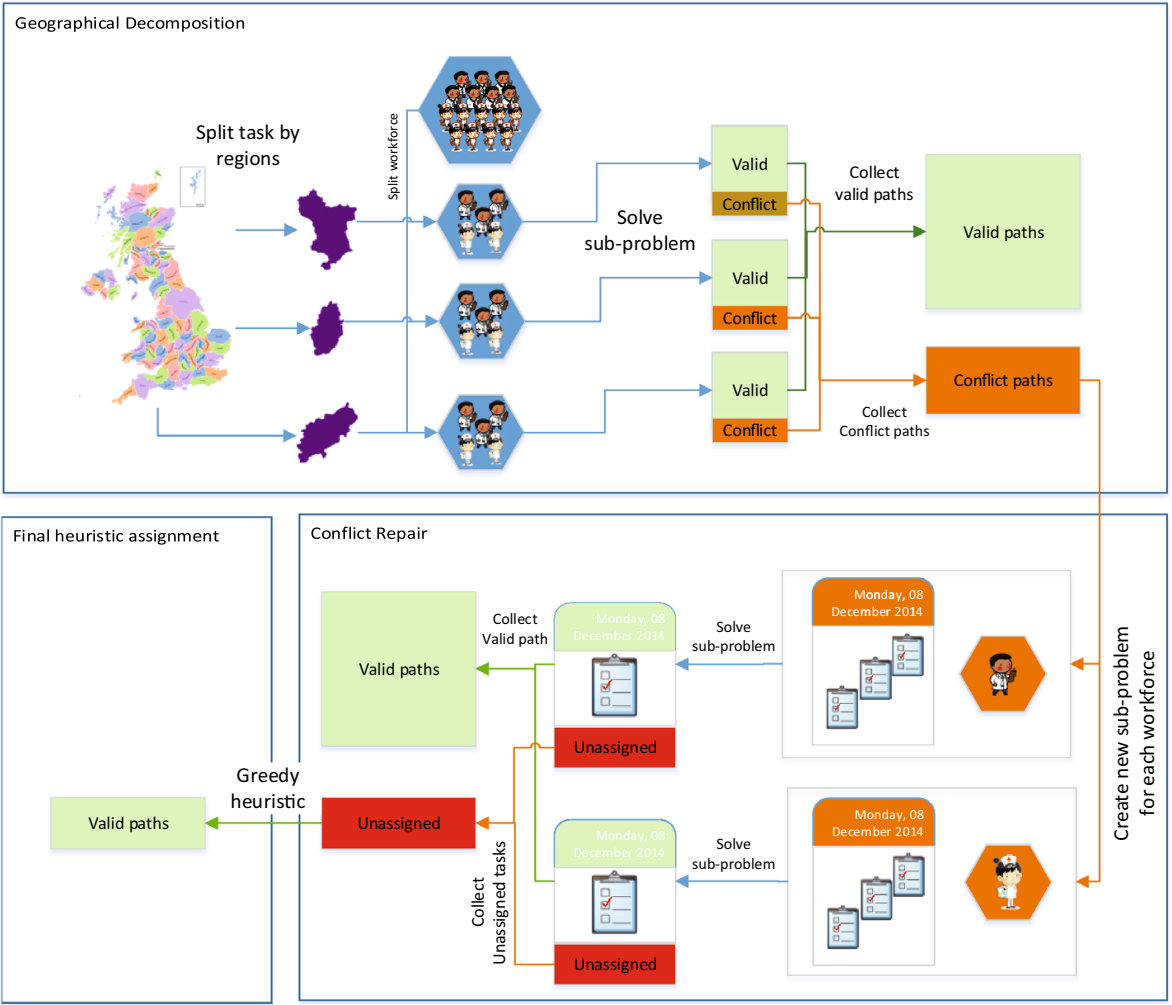
The Geographical Decomposition with Conflict Repair (GDCR) Approach

Figure 2 shows the outline of the GDCR. The upper rectangle in the figure illustrates the geographical decomposition, the lower right rectangle illustrates the conflicting assignments repair and the lower left rectangle illustrates the heuristic assignment. In summary, GDCR decomposes a problem instance by geographical regions and solves each sub-problem but not preventing conflicting assignments. Then, conflicting assignments are repaired which may result in some unassigned tasks. Finally, a heuristic assignment algorithm is used to deal with unassigned tasks. Each of these stages is further explained later in this paper.
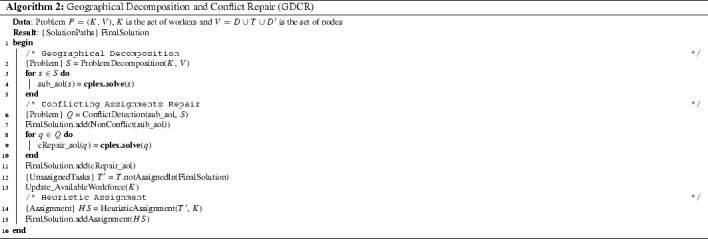


Algorithm 2 outlines the GDCR method which takes a problem instance and generates a solution by assigning paths to workforce. The algorithm shows the three stages executed in sequence: geographical decomposition (lines 2–5), conflicting assignments repair (lines 6–10) and heuristic assignment (line 14). We now proceed to describe these three processes in Sects. 5.1, 5.2 and 5.3 respectively. Each sub-problem is defined by the MIP model presented in Sect.2 [not including constraints (15) and (16)] and solved to optimality by the MIP solver.

### Geographical decomposition

As illustrated in Fig. 2, the geographical decomposition stage decomposes the problem into several smaller sub-problems. This is done exactly as in GDCA, i.e. the sub-problems are defined by the geographical regions. Then, each sub-problem contains the task nodes located in the same geographical region and workers who are available to work in that region. Each sub-problem also contains the start/end location nodes. Algorithm 3 outlines this stage. The set of tasks are partitioned (line 2) and workforce selected for the tasks in each partition $T_n$ (line 4). The sub-problems are generated by *subproblem_builder* which basically collects data for the sub-problem.
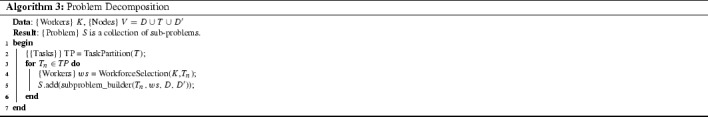


#### Tasks partition

The size of each sub-problem is restricted by the task partitioning process within the geographical decomposition stage, as shown in Algorithm 4. This process takes the set of task nodes *T* and returns a partition set ${ TP}$ with no partition element larger than *subProblemSize*. All tasks located in each region $a \in A$ are added to the subset $T_a$ (line 5). If the size of subset $T_a$ is larger than the maximum allowed sub-problem size (20 locations), then the subset of tasks $T_a$ is further partitioned by uniformly distributing the tasks over the second level subsets *W* (line 10). This second partitioning layer is crucial to control the size of sub-problems so that they can be solved to optimality in practical time by the MIP solver.
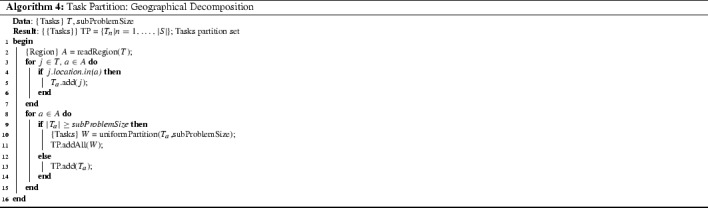


#### Workforce selection

We remind the reader that each worker is usually associated to more than one geographical region, hence the workforce set cannot be partitioned like the tasks. A worker can be used in multiple sub-problems. This is exactly like in GDCA but in that approach, the additional constraints (15) and (16) prevent conflicting assignments. Since these constraints are not used in this GDCR approach, solving each of the $s_i$ sub-problems independently may provoke a worker to be assigned to different tasks at the same time in different sub-problems. Hence, the conflicting assignments repair procedure described next is implemented.

### Conflicting assignments repair

This process takes the solution from solving each of the $s_i \in S$ sub-problems and identifies conflicting assignments to form conflict sub-problems. For each path $\Phi _i$ in the solution of sub-problem $s_i$, the algorithm searches for all the conflicting paths in the other sub-problem solutions. A conflicting path is any other path $\Phi _j$ that uses the same worker as $\Phi _i$. Then, if conflicting paths exist for a worker, they are removed from the sub-problem solutions and put together in a conflict sub-problem. Each conflict sub-problem is defined by the MIP model presented in Sect. 2 and corresponds to exactly one worker and all tasks that were in the set of conflicting paths. Each conflicting sub-problem is then solved with the MIP solver as shown in line 9 of Algorithm 2. Solving a conflicting sub-problem gives a single valid path for the worker but perhaps with some unassigned tasks due to the optimization process. The heuristic assignment process described next seeks to incorporate these unassigned tasks into the overall solution.

### Heuristic assignment

At this stage, the set of unassigned tasks $T'$ that result from the conflicting assignments repair procedure are tackled with a heuristic assignment method. This is a simple greedy approach that seeks to assign tasks to the worker that incurs the least cost. Algorithm 5 outlines this procedure. Basically, for each unassigned task $j \in T'$ it seeks the most cost efficient worker $k \in K$ that can take the task without provoking a conflicting assignment. This may result in the assignment not respecting the worker’s predefined geographical regions, but as explained earlier in this paper, this is a soft requirement in our HHC scenarios.
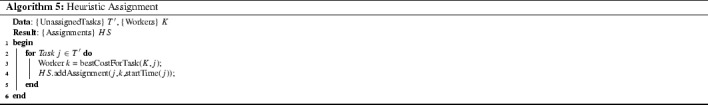


### Experimental study on the stages of GDCR

We now present experimental results to investigate how the three stages in the GDCR method contribute to generating a final solution to the whole problem instance. For this, we scrutinize the proportion of assigned tasks, travelling distance and computation time that each of these stages deals with in producing a solution.

**Fig. 3:**
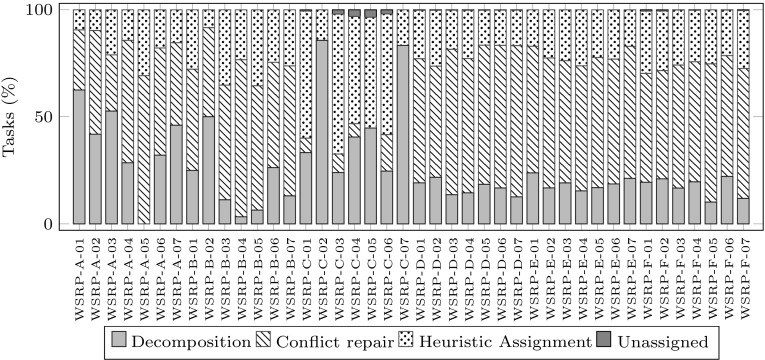
Proportion of tasks assigned in each stage of GDCR. *Each bar* shows for a problem instance, the percentage of tasks assigned by each stage: decomposition, conflict repair and heuristic assignment. In very few cases there are tasks left unassigned after the three stages are completed

Figure 3 shows the proportion of tasks assigned in each of the three stages. Each of the 42 stacked bars corresponds to 100 % of the number of tasks in the corresponding problem instance. Each stacked bar has four parts: decomposition, conflicts repair, heuristic assignment and unassigned tasks. Each part indicates the proportion of tasks assigned in the corresponding stage of GDCR. On average, the proportion of tasks assigned by decomposition, conflicts repair and heuristic assignment were 26.34, 47.50 and 25.82 % respectively. Only 0.34 % of the tasks were left unassigned. In general, the conflicts repair stage achieves the largest proportion of successful assignments, except for instances WSRP-C. In these instances most tasks have a long duration of 6–9 h. Therefore, it is likely that workers could take only one task in the solution to each sub-problem. Therefore, the conflicting assignments repair stage was less successful in solving conflicting sub-problems.

**Fig. 4:**
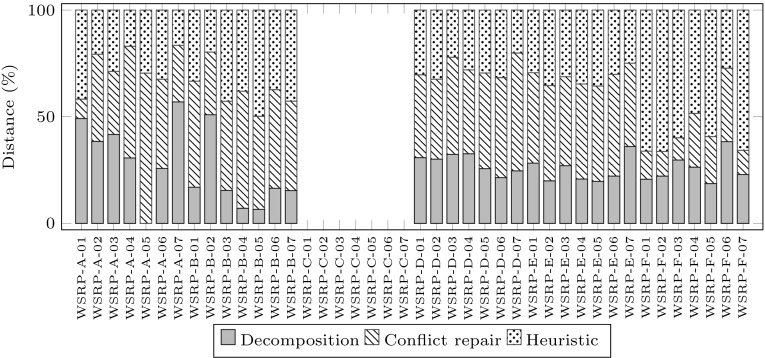
Proportion of travelling distance generated in each stage of GDCR. *Each bar* shows for a problem instance, the percentage of travelling distance in the portion of path generated by each stage: decomposition, conflict repair and heuristic assignment

Figure 4 shows the proportion of travelling distance generated in each of the three stages. Each of the 42 stacked bars corresponds to the total travelling distance in the final solution to the corresponding problem instance. Note that there is no bar for WSRP-C instances because no travelling between locations takes place in these solutions. Each stacked bar has three parts: decomposition, conflicts repair and heuristic assignment. Each part indicates the proportion of travelling distance generated in each stage. These are 26.36 % for decomposition, 37.64 % for conflicts repair and 36.0 % for heuristic assignment.

**Fig. 5:**
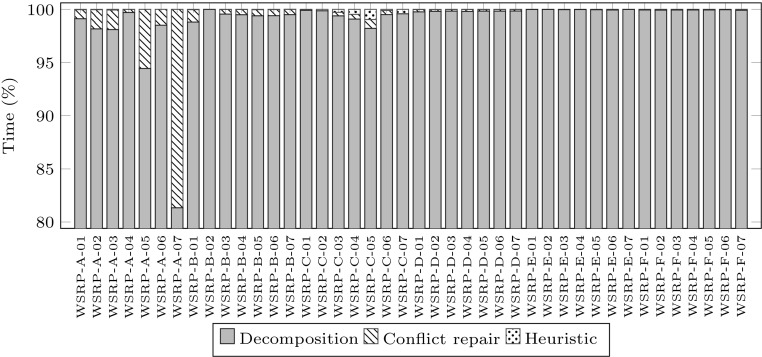
Proportion of computation time used in each stage of GDCR. *Each bar* shows for a problem instance, the percentage of computation time used by each stage: decomposition, conflict repair and heuristic assignment

Figure 5 shows the proportion of computational time used by each of the three stages. Each of the 42 stacked bars corresponds to the total computational time spent to produce a solution to the corresponding problem instance. For better visualization, the y-axis only shows from 80 to 100 %. Each stacked bar has three parts: decomposition, conflicts repair and heuristic assignment. Each part indicates the proportion of computational time spent in each stage. While the geographic decomposition stage consumes most of the time. This is because the sub-problems generated in this stage are much larger than those generated in the conflicts repair stage. On the other hand, heuristic assignment is very quick, particularly for instances WSRP-D, WSRP-E and WSRP-F, taking less than 0.1 % of the total computational time.

One way to shorten the computational time of the decomposition stage would be to reduce the size of the decomposition sub-problems. However, this would mean more conflicting paths and hence larger conflicting sub-problems to tackle with the conflicts repair stage. It would also mean more unassigned tasks to tackle with the heuristic assignment stage. Hence, in the next section we propose an iterative decomposition and conflict repair approach.

## Repeated decomposition and conflict repair (RDCR)

An improved method called Repeated Decomposition and Conflict Repair (RDCR) is presented here aimed at reducing the computational time spent in the geographical decomposition step and improving the overall solution quality. While the GDRC method described above sets the sub-problem size at 20 locations, RDCR sets it at 20 tasks (a reduction in size because one location can be associated to multiple tasks). Also, RDCR uses the decomposition and conflicts repair stages repeatedly until no assignment can be made. The aim is to have a higher utilization of the MIP solver instead of relying in the heuristic assignment stage. Moreover, we investigate different criteria for the decomposition besides geographical regions aiming to further reduce the computational time.

**Fig. 6:**
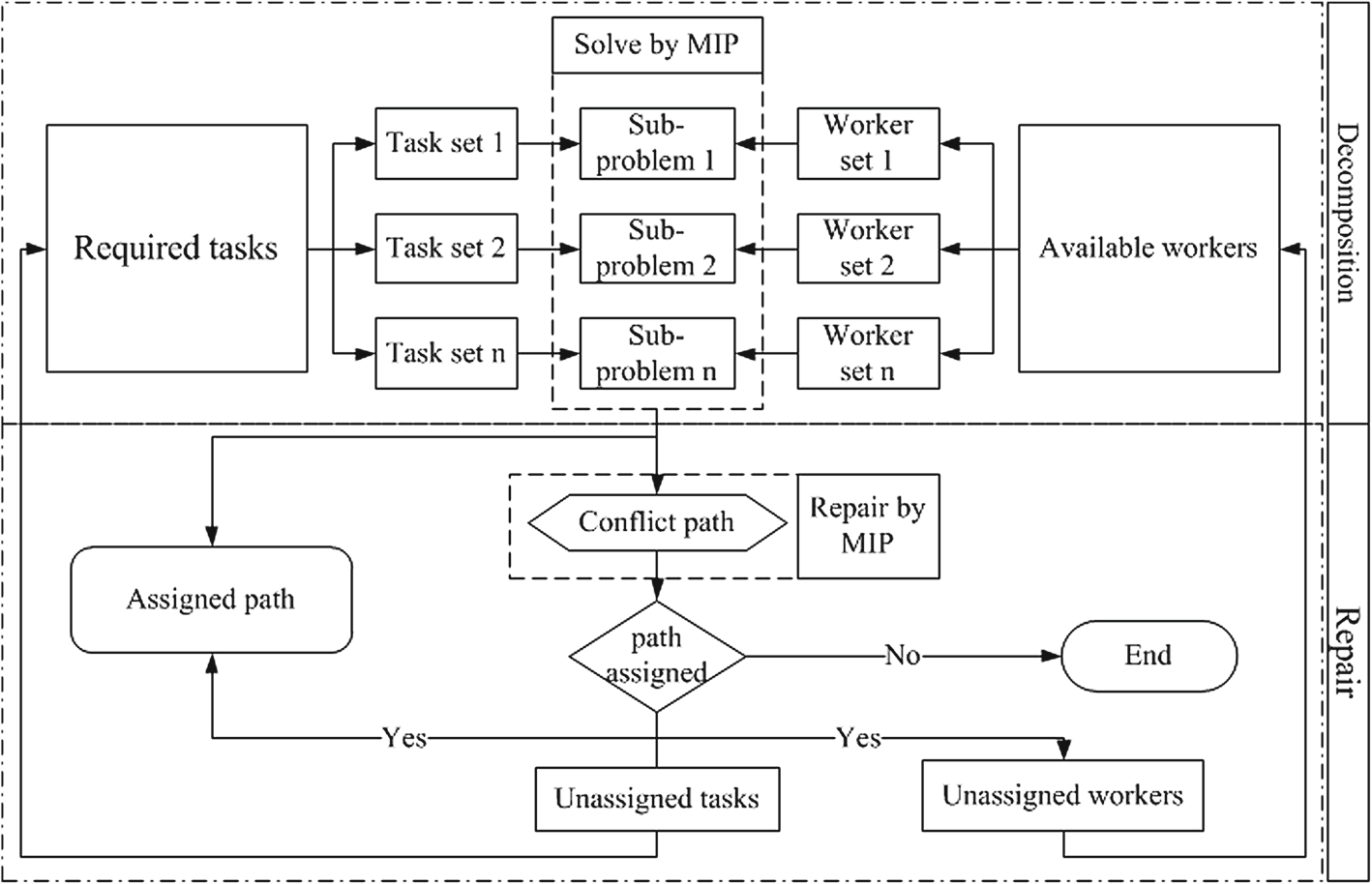
Overview of Repeated Decomposition and Conflict Repair method

Figure 6 shows the outline of the RDCR in two parts. The upper part is the problem decomposition and the lower part is the conflicts repair, these two are used iteratively to find an overall solution. Algorithm 6 outlines the RDCR method. Compared to GDCR, the RDCR approach drops the heuristic assignment stage and relies on the problem decomposition and conflicts repair stages only. Details if the RDCR method are explained in the subsections below.
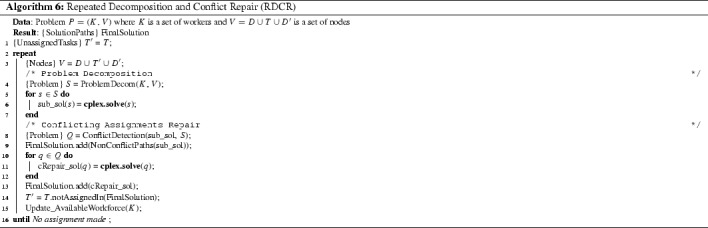


### Problem decomposition

As before, we split the problem into several smaller sub-problems. The outline of this process remains like in GDCR, see Algorithm 3. However, here we use different approaches for task partition and workforce selection as describe next.

#### Tasks partition

Besides partitioning tasks based on their location, we also use a clustering algorithm, namely *k*-medoids, to define the partition based on distance between locations. The *k*-medoids algorithm works in the same way as the *k*-means algorithm (Park and Jun 2009). It finds *k* clusters based on the distance between the items. For better computational efficiency when tackling the sub-problems with the MIP solver, it is also desirable that the clusters are of similar size. Therefore, we propose three rules for tasks partitioning as described next.

*Location based with uniform partition (LBU)* This procedure partitions tasks according to their location while also aiming to limit the size of each subset. The procedure is shown in Algorithm 7. First, tasks are ordered by location into *tasksList* and processed one at a time. Task *j* in *tasksList* is allocated to subset $T_n$ if the task has the same location as any task already in the subset or if the maximum size of the subset has not been reached. If task *j* is not allocated to an existing subset then a new subset is created. We set *subproblemSize* to 20 tasks. Since most of our 42 HHC instances have locations with no more than 5 tasks, this LBU procedure mostly generates subsets within the size limit or at most a handful of more tasks.
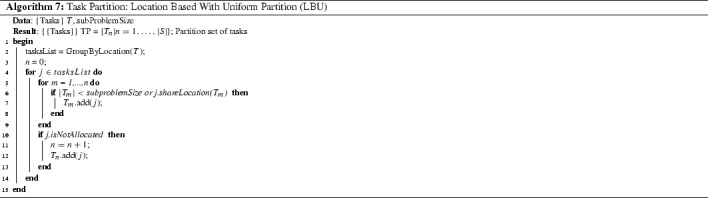


*Region based with k-medoids clustering partition (RBK)* This procedure partitions tasks according to geographical regions and then splits too large subsets (regions with a high density of tasks) using the *k*-medoids clustering algorithm. The result is a set of sub-problems where tasks within the same sub-problem share the same region and are separated by short travelling distances. The procedure is shown in Algorithm 8. First, tasks are partitioned by geographical regions into *A* and each subset $T_a$ is processed one at a time. Then, the *k*-medoids clustering algorithm is applied to those subsets that have a size larger than *subproblemSize* (20 tasks). The clustering algorithms seeks to minimize travelling distance between tasks in the same cluster and the number of clusters size is calculated by dividing the number of tasks in the subset $T_a$ by *subProblemSize*.
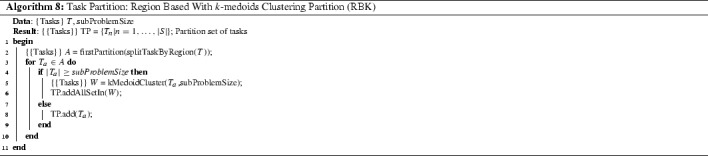


*Skills based with k-medoids clustering partition (SBK)* This procedure is a variant of RBK explained above. The only difference is that the first partitioning level is based on the skills required by tasks instead of by geographical regions. Then, in Algorithm 8, we replace *splitTaskByRegion* at line 2 by *splitTaskBySkill*. The first partitioning level gives subsets with tasks that require the same set of skills. This helps to group tasks that may require specialist workers. Such workers with specific skills are usually low in numbers but may be required to cover tasks in a wide area. The second partitioning level using *k*-medoids clustering is applied next to reduce the size of larger subsets, those including tasks that require more general skills.

#### Workforce selection

We propose three workforce selection methods as described next, to complete the sub-problems in RDCR. The aim is to select a not too large subset of workers that are suitable for the tasks already in the sub-problem.

*Best fitness selection (BF)* This procedure finds a set of best workers, where each worker is one of the best candidates for each task in the subset. For each task *j* in a subset $T_n$ we identify the best worker by partially computing the objective function (14). For this, the assignment of each worker to task *j* is evaluated by computing three components of the objective function: monetary cost, preferences penalty, and soft constraints penalty. The worker must also have the required skills for the task. If the best worker identified for task *j* has been already selected for another task in the same $T_n$, then the next best worker is selected and so on. This selection method guarantees that all tasks can be assigned unless there is no worker with the required skills for the task. The resulting sub-problem has at most one worker for each task.

*Best average fitness selection (AF)* This procedure finds a set of good average workers, where each worker is a good candidate for all the tasks in the subset. Similarly to the BF procedure, for each task $j \in T_n$ and each worker, we partially compute the objective function (14). But instead of selecting the best worker for the task, we select the next best average worker. Workers are listed in decreasing order of their average partial objective function value considering all tasks in the subset $T_n$. The next available best average worker is selected for the subset until we have the same number of workers as tasks in the subset.

*Workers suitability selection (WS)* This procedure finds a set of suitable workers, based on skills and locations, for all the tasks in the subset. All workers that have the required skills and location availability for at least one task in the subset are selected for the subset. This selection procedure results in larger number of workers for each sub-problem, which would demand more computational time when solving the sub-problems but could result in higher quality solutions.

#### Repeated sub-problems solving

Solving the sub-problems with the MIP solver is carried out iteratively until a final solution with a set of valid paths (with no conflicting assignments) is obtained. As before, the sub-problems generated with the above procedures are defined by the MIP model presented in Sect. 2 [not including constraints (15) and (16)]. There are no conflicting assignments between the paths in the same sub-problem solution, but there might be conflicting assignments between paths in different sub-problems. Instead of using the heuristic assignment procedure as in GDCR, only the MIP solver is used in an iterative process of generating sub-problems and solving them (repairing the solution). In our experiments, small instances required 2 or 3 iterations of RDCR while larger instances required 5–6 or a few more iterations. Always the first iteration was the most time consuming with the later ones (repeated repairs) being much faster.

### Experimental study on the sub-problem generation methods

We now present experimental results to investigate how the three procedures to partition tasks (LBU, RBK and SBK) and the three procedures to select workforce (BF, AF and WS) contribute to generating a final solution to the whole problem instance. The nine combinations are tested on the 42 problem instances and results are collected in terms of the solution quality and computation time. In the results presented here, LBU-BF denotes location based with uniform tasks partition followed by best fitness workforce, similar naming convention is used for the other sub-problem generation procedures.

**Fig. 7:**
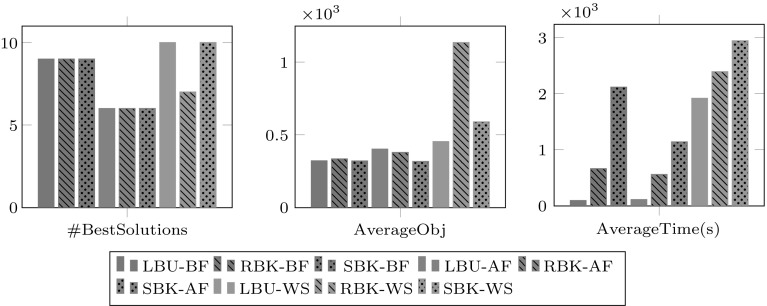
Overall results using the nine decomposition procedures within RDCR on the 42 problem instances. The sub-figure on the *left* shows the number of best known solutions found with each procedure. The sub-figure in the *middle* shows the average objective value obtained with each procedure. The sub-figure on the *right* shows the average computational time in seconds when using each procedure

Figure 7 presents the summary of results comparing the nine sub-problem generation methods. From left to right, the figure shows the number of best solutions (#BestSolutions), average objective value (AverageObj) and average computational time (AverageTime) in seconds. Each bar in each sub-figure shows the results obtained for all the 42 instances when using one particular sub-problem generation method within RDCR.

In terms of number of best solutions, LBU-WS and SBK-WS achieve the highest number of best solutions (10 instances), followed by LBU-BF, RBK-BF and SBK-BF with 9 best solutions each. In terms of average objective value, eight of the methods gave very competitive results while only RBK-WS showing considerable lower performance. In terms of average computational time, the figure seems to indicate that the LBU tasks partitioning procedure combined with either BF or AF workforce selection are the fastest methods. The next fastest ones are the RBK tasks partitioning procedure combined with either BF or AF. The three methods using the WS workforce selection method are the most time consuming. As mentioned before, we were expecting this to be the case as selecting all suitable workers increases the sub-problem size. However, we though that this workforce selection method would result is better solutions but this was not the case as it can be seen in the other sub-figures. We should note that there was a time limit set for solving each sub-problem of 30 seconds per task.

We also conducted a statistical analysis using the non-parametric Friedman’s ANOVA test to determine any statistical significant difference, in terms of solution quality and computation time, between the sub-problem generation methods. We used SPSS (Field 2013) and set the main significant level of the test at 0.05. Based on the results of this study we selected the LBU-BF method to be used within RDCR. Detailed results of this study are presented in the “Appendix”.

**Table 2:** Comparison between GDCR and RDCR on the same decomposition rule (LBU-BF)

Instance	GDCR		RDCR		$\%\hbox {Diff}$	Instance	GDCR		RDCR		$\%\hbox {Diff}$
	%Heur	Obj	#Iter	Obj			%Heur	Obj	#Iter	Obj	
WSRP-A-01	6.25	4.06	2	**4**.**00**	1.50	WSRP-B-01	19.44	1.88	2	**1**.**76**	6.28
WSRP-A-02	6.45	2.98	2	**2**.**93**	1.71	WSRP-B-02	0.00	**1**.**80**	1	**1**.**80**	0.00
WSRP-A-03	21.05	7.14	2	**6**.**86**	3.92	WSRP-B-03	35.21	2.08	3	**1**.**85**	11.15
WSRP-A-04	10.71	2.01	2	**1**.**95**	3.29	WSRP-B-04	13.33	2.13	2	**2**.**12**	0.51
WSRP-A-05	0.00	**2**.**42**	1	**2**.**42**	0.00	WSRP-B-05	30.65	3.11	3	**2**.**98**	4.09
WSRP-A-06	14.29	3.70	2	**3**.**56**	3.83	WSRP-B-06	21.05	1.82	2	**1**.**75**	3.56
WSRP-A-07	0.00	**3**.**71**	1	**3**.**71**	0.00	WSRP-B-07	32.79	2.59	2	**2**.**03**	21.5
WSRP-C-01	22.60	172.38	3	**131**.**57**	23.68	WSRP-D-01	25.97	214.45	4	**205**.**26**	4.28
WSRP-C-02	0.00	**3**.**15**	1	**3**.**15**	0.00	WSRP-D-02	29.46	204.52	4	**198**.**78**	2.81
WSRP-C-03	26.67	161.02	3	**159**.**44**	0.98	WSRP-D-03	23.11	208.49	4	**207**.**87**	0.30
WSRP-C-04	0.00	**13**.**08**	1	**13**.**08**	0.00	WSRP-D-04	28.32	**209**.**00**	4	211.79	$-$1.34
WSRP-C-05	6.90	15.26	2	**15**.**25**	0.12	WSRP-D-05	20.07	186.81	4	**183**.**73**	1.65
WSRP-C-06	40.51	**193**.**12**	3	196.90	$-$1.96	WSRP-D-06	19.69	200.96	4	**198**.**89**	1.03
WSRP-C-07	0.00	**4**.**30**	1	**4**.**30**	0.00	WSRP-D-07	19.94	**200**.**45**	4	202.85	$-$1.20
WSRP-E-01	18.64	**3**.**30**	4	5.19	$-$57.1	WSRP-F-01	30.21	3392.52	5	**2149**.**79**	36.6
WSRP-E-02	22.27	**1**.**87**	4	3.22	$-$72.2	WSRP-F-02	30.47	**2213**.**27**	5	2505.38	$-$13.2
WSRP-E-03	25.05	**2**.**40**	4	4.23	$-$76.6	WSRP-F-03	25.13	711.43	4	**703**.**85**	1.07
WSRP-E-04	26.17	2.47	4	**1**.**79**	27.5	WSRP-F-04	28.61	1450.50	4	**1447**.**87**	0.18
WSRP-E-05	22.73	**3**.**89**	4	7.26	$-$86.6	WSRP-F-05	26.55	319.47	5	**314**.**84**	1.45
WSRP-E-06	24.61	2.57	4	**2**.**30**	10.1	WSRP-F-06	25.86	755.98	4	**742**.**35**	1.80
WSRP-E-07	17.85	**4**.**31**	4	7.71	$-$78.8	WSRP-F-07	25.45	3619.02	5	**3604**.**45**	0.40

Table presents percentage of heuristic assignment made in GDCR, objective value from using GDCR, the number of iterations used in RDCR, objective value from using RDCR and percentage differences between the two solutions

Bold text presents better solution

Positive percentage difference refers to the case that RDCR provides better solution

Negative percentage difference refers to the case that GDCR provides better solution

### Experimental study on solution improvement in RDCR

This section presents experimental results to assess the improvements achieved in RDCR from the repeated use of the MIP solver. For this, both GDCR and RDCR are run using the same decomposition rule LBU-BF and their performance compared. Table 2 compares the solutions obtained by the two methods on the 42 HHC instances from the six behnchmark scenarios. Column GDCR %Heur shows the percentage of assignments to be made by the heuristic assignment step in GDCR. In RDCR, these assignments are tackled by the second and following iterations (remeber that the first decomposition and conflict repair of both algorithms provides the same solution). Column RDCR #Iter shows the number of iterations used in RDCR. Columns GDCR Obj and RDCR Obj show the objective value for each solution found by GDCR and RDCR, respectively. Finally, column %Diff shows the difference between the two solutions, where a positive value indicates that the RDCR solution is better and a negative value indicates the opposite.

The results in Table 2 show that the first decomposition and conflict repair steps find a solution to the whole problem for six instances: WSRP-A-05, WSRP-A-07, WSRP-B-02, WSRP-C-02, WSRP-C-04, and WSRP-C-07, i.e. both GDCR and RDCR produce the same solution quality in these cases. The table also shows that RDCR produces better solutions that GDCR for 28 instances while GDCR is better in 9 instances. This is a clear indication that using the MIP solver iteratively in RDCR results in increased solution quality compared to the using the heuristic assignment algorithm in GDCR. However, focusing in instance set WSRP-E, we can see that GDCR clearly outperforms RDCR in 5 of the 7 instances by a margin between 57 and 86 %. The improvement achieved by RDCR over GDCR in the other two instances, WSRP-E-04 and WSRP-E-06, is by a margin of 27 and 10 %, respectively. The instance set WSRP-E is a scenario where workers are mostly suitable to make every visit. The iterative approach in RDCR might not work well in such conditions of over-fitting workforce because splitting the decisions on assignments across sub-problems might prevent RDCR to achieve the overall better solution. Since GDCR tackles the rest of the assignments in one step, this might help the method to perform better in these cases. Therefore, we can conclude that RDCR is better than GDCR in general but it is also clearly outperformed by GDCR in most of the instances in group WSRP-E.

## Experimental study on the decomposition methods

This section presents results from the experiments conducted to compare the overall performance of the three decomposition methods variants described in this paper, geographical decomposition with conflict avoidance (GDCA), geographical decomposition with conflict repair (GDCR) and repeated decomposition and conflict repair (RDCR). Results obtained with these three approaches are compared to solutions obtained from the greedy heuristic assignment approach described in Algorithm 5, solutions produced by a human planner and optimal solutions (when available) from the MIP solver.

**Fig. 8:**
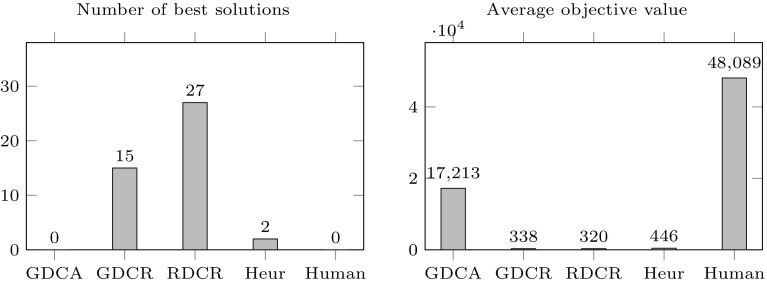
Number of best known solutions (*left* sub-figure) and average objective function (*right* sub-figure) of GDCA, GDCR, RDCR, heuristic assignment (Heur) and solution from a human planned (Human). A lower objective value presents a better result

First, overall results considering all 42 problem instances are presented in Fig. 8. The graph on the left shows the number of best solutions obtained by each method. The graph on the right shows the average objective function value (the lower the better) obtained by each method. It can be observed that in terms of number of best solutions, RDCR is the best method producing best known solutions for 27 of the 42 instances. The second best is GDCR with 15 best solutions. The heuristic produced only 2 best solutions while GDCA and the human planner did not produce best solutions. In terms of the average objective function value, a similar trend can be observed with RDCR at the top followed by GDCR, the heuristic, GDCA and the human planner respectively.

Now, more detailed results are presented for each of the 42 problem instances. Table 3 shows results for the objective function value. These results also include the optimal solution, only available for some small instances, obtained by solving the instance as a whole using the MIP solver. The best results for each instance are shown in bold. The table shows that for most instances in which the optimal solution is available, none of the methods is able to find an optimal solution. The exceptions are RDCR finding optimal solutions for instances WSRP-A-05, WSRP-A-07, WSRP-C-02 and WSRP-C-07, and GDCR finding optimal solutions for instances WSRP-B-02, WSRP-C-02 and WSRP-C-07. For the small instances where no optimal solution is available (WSRP-C-01, WSRP-C-03 and WSRP-C-06), RDCR found the best known solutions. For the 21 larger instances, RDCR obtained best know solutions for 12 of them, GDCR obtained best know solutions for 9 of them (a tie with RDCR in one instance), and the heuristic obtained best know solutions for 2 of them (a tie with RDCR in one instance).

**Table 3:** Solution objective values for the optimal solution, and obtained by GDCA, GDCR, RDCR, heuristic assignment (Heur) and human planner on the 42 instances

Instance	Optimal	GDCA	GDCR	RDCR	Heur	Human	Instance	GDCA	GDCR	RDCR	Heur	Human
WSRP-A-01	**3**.**49**	5.65	4.48	4.00	5.46	307	WSRP-D-01	496	210	**205**	236	17,386
WSRP-A-02	**2**.**49**	4.53	3.36	2.93	4.42	75.3	WSRP-D-02	373	206	**199**	244	13,830
WSRP-A-03	**3**.**00**	10.6	4.93	6.86	7.41	68.4	WSRP-D-03	3213	229	**208**	221	26,919
WSRP-A-04	**1**.**42**	3.09	2.49	1.95	2.36	93.2	WSRP-D-04	419	219	**212**	223	16,677
WSRP-A-05	**2**.**42**	3.54	3.12	**2**.**42**	3.15	24.5	WSRP-D-05	244	202	**184**	189	33,705
WSRP-A-06	**3**.**55**	3.74	3.62	3.56	5.67	24.6	WSRP-D-06	1411	223	**199**	**199**	23,869
WSRP-A-07	**3**.**71**	4.81	4.07	**3**.**71**	7.10	27.7	WSRP-D-07	753	218	203	**197**	22,794
WSRP-B-01	**1**.**70**	1.79	1.89	1.76	2.87	200	WSRP-E-01	33.0	**3**.**69**	5.19	6.27	180,633
WSRP-B-02	**1**.**75**	1.89	**1**.**75**	1.80	2.83	1.94	WSRP-E-02	26.0	**2**.**21**	3.22	4.83	78,012
WSRP-B-03	**1**.**72**	2.06	1.89	1.85	2.97	692	WSRP-E-03	29.0	**1**.**23**	4.23	8.27	61,624
WSRP-B-04	**2**.**07**	2.21	2.13	2.12	3.01	130	WSRP-E-04	28.5	**1**.**79**	**1**.**79**	4.30	101,369
WSRP-B-05	**1**.**82**	4.74	2.54	2.98	2.88	623	WSRP-E-05	270	**3**.**76**	7.26	8.25	32,075
WSRP-B-06	**1**.**62**	2.52	1.75	1.75	2.91	112	WSRP-E-06	24.6	**2**.**30**	2.30	5.43	80,479
WSRP-B-07	**1**.**79**	4.06	2.94	2.03	3.44	474	WSRP-E-07	428	**4**.**72**	7.71	5.68	142,485
WSRP-C-01	N/A	905	133	**131**	185	29,642	WSRP-F-01	64,305	2740	**2150**	2810	89,383
WSRP-C-02	**3**.**15**	3.61	**3**.**15**	**3**.**15**	4.86	6.41	WSRP-F-02	73,291	**2482**	2505	3235	117,274
WSRP-C-03	N/A	1186	196	**159**	170	24,295	WSRP-F-03	115,235	707	**704**	1619	141,427
WSRP-C-04	**11**.**15**	81.3	23.1	13.1	16.5	997	WSRP-F-04	102,994	1453	**1448**	1958	110,104
WSRP-C-05	**12**.**34**	68.9	22.4	15.3	17.6	752	WSRP-F-05	101,438	**297**	315	1752	336,684
WSRP-C-06	N/A	3102	198	**197**	251	11,486	WSRP-F-06	76,007	747	**742**	862	146,456
WSRP-C-07	**4**.**30**	5.29	**4**.**30**	**4**.**30**	5.82	10.7	WSRP-F-07	176,541	3610	**3604**	4239	176,524

Optimal solution is not found on instances marked as N/A

Bold text presents better solution

Table 4 shows results for the computational time in seconds. Note that the time spent in generating the human planner solution is not available. As expected, the computational time spent in finding the optimal solution (when available) is usually the highest, particularly for instance WSRP-B-03. It is clear that the heuristic is the fastest method for all problem instances taking a fraction of a second in most cases and less than 3 seconds in the others. The second fastest times are shown in bold and we can see than for almost all problem instances RDCR is the fastest method. The exception is instance WSRPA-03 for which GDCA is the fastest method. An overall comparison of the three decomposition methods proposed here, shows that RDCR is the fastest, followed by GDCR and GDCA.

**Table 4:** Computational time in seconds for optimal solution, GDCA, GDCR, RDCR and heuristic assignment (Heur) on the 42 instances

Instance	Optimal	GDCA	GDCR	RDCR	Heur	Instance	GDCA	GDCR	RDCR	Heur
WSRP-A-01	7	3.71	3.76	**2**.**53**	<.1	WSRP-D-01	1060	579	**109**	0.18
WSRP-A-02	8	3.58	3.35	**2**.**39**	<.1	WSRP-D-02	1192	706	**109**	0.14
WSRP-A-03	14	**3**.**70**	4.69	5.17	<.1	WSRP-D-03	1209	1024	**127**	0.18
WSRP-A-04	5	2.88	2.29	**1**.**87**	<.1	WSRP-D-04	3005	785	**127**	0.17
WSRP-A-05	1	1.77	1.28	**0**.**76**	<.1	WSRP-D-05	1307	907	**118**	0.18
WSRP-A-06	5	2.42	2.80	**1**.**77**	<.1	WSRP-D-06	1222	1064	**130**	0.2
WSRP-A-07	1	1.64	1.55	**0**.**70**	<.1	WSRP-D-07	1362	1133	**142**	0.23
WSRP-B-01	21	8.07	6.96	**4**.**67**	<.1	WSRP-E-01	8408	7676	**93**.**51**	0.19
WSRP-B-02	2	4.29	3.36	**0**.**79**	<.1	WSRP-E-02	12,448	9806	**86**.**84**	0.18
WSRP-B-03	6003	32.86	37.97	**10**.**51**	<.1	WSRP-E-03	20,747	11,872	**101**	0.22
WSRP-B-04	25	15.25	12.19	**2**.**63**	<.1	WSRP-E-04	15,190	8758	**71**.**57**	0.18
WSRP-B-05	585	25.35	23.22	**8**.**31**	<.1	WSRP-E-05	32,619	9510	**98**.**63**	0.25
WSRP-B-06	184	24.11	21.80	**8**.**78**	<.1	WSRP-E-06	24,212	9121	**65**.**60**	0.15
WSRP-B-07	300	23.64	24.44	**8**.**14**	<.1	WSRP-E-07	51,057	13,884	**107**	0.27
WSRP-C-01	N/A	212	224	**25**.**50**	0.34	WSRP-F-01	3446	1788	**250**	1.00
WSRP-C-02	6	0.57	0.63	**0**.**12**	<.1	WSRP-F-02	1111	1730	**251**	1.20
WSRP-C-03	N/A	26.33	27.84	**18**.**22**	0.26	WSRP-F-03	4555	1908	**342**	1.61
WSRP-C-04	90	3.09	3.84	**1**.**07**	0.11	WSRP-F-04	4219	7060	**360**	1.67
WSRP-C-05	55	1.05	1.91	**0**.**71**	<.1	WSRP-F-05	6157	3437	**390**	1.91
WSRP-C-06	N/A	47.05	49.77	**24**.**94**	0.19	WSRP-F-06	9696	7204	**442**	1.91
WSRP-C-07	1	0.24	0.23	**0**.**11**	<.1	WSRP-F-07	3833	1847	**422**	2.46

Time presents in seconds. Optimal solution is not found on instances marked as N/A

Bold text presents the second best computational time as the fastest time always belongs to heuristic assignment

Then, considering both solution quality and computational efficiency, we can conclude that the RDCR method is the best one overall as it ranked first in terms of solution quality and second in terms of computational time. Among the three decomposition method variants, RDCR is the fastest in terms of computational time while still making all task assignments with the MIP solver. The reduction in computational time compared to GDCR and GDCA is mainly from the reduced sub-problem size achieved in the decomposition step. Also, the repeated process of generating and solving sub-problems is able to generate solutions of higher quality. RDCR also shows that harnessing the power of the MIP solver produces better results than the heuristic algorithm. It seems that although GDCR produces good results, using the assignment heuristic brings a limitation on the quality of solutions obtained compared to RDCR.

We also conducted a statistical analysis using the non-parametric Friedman’s ANOVA test to determine any statistical significant difference in performance between the methods. Based in the results from this study we conclude that in terms of objective function value, the better ranked methods are RDCR and GDCR and in terms of the computational time the better ranked methods are the heuristic and RDCR. Detailed results of this study are presented in the “Appendix”.

## Conclusions and future work

This paper has investigated decomposition techniques combining mixed-integer programming (MIP) solvers and heuristics to tackle real-world instances of the home healthcare planning (HHC) problem. The goal in this problem is to plan visits by workers (e.g. nurses and care workers) to patients at their homes in order to carry out some healthcare related tasks. HHC problems involve both scheduling and routing and it has been shown in the literature that these are very difficult problems to solve. This paper proposes effective approaches to harness the power of modern MIP solvers by means decomposition in order to produce high quality solutions in practical computation time. Experiments were conducted using 42 instances from 6 different real-world HHC scenarios provided by our industrial partner, a provider of workforce management software as a service.

The paper investigated three variants of the problem decomposition approach at the centre of this research. The overall strategy is as follows. First, to generate sub-problems by some decomposition method. Second, to solve the sub-problems with an MIP solver. Third to integrate the sub-problem solutions into a valid solution to the whole problem. The decomposition techniques proposed here differ in two main aspects: (1) the method to decompose the problem into sub-problems and (2) the method to deal with conflicting assignments (a worker assigned to more than one task at a given time).

The first decomposition method is called Geographical Decomposition with Conflict Avoidance (GDCA). This approach decomposes the problems into sub-problems by splitting tasks according to geographical regions defined by the practitioner. After solving each sub-problem, this method only considers the suitable workforce not used in the previous solved sub-problems so that conflicting assignments are prevented. The main issue with this approach is that the sequence in which sub-problems are solved influences the overall solution quality obtained.

The second decomposition method is called Geographical Decomposition with Conflict Repair (GDCR). This approach differs from GDCA in that the whole suitable workforce is used when solving each sub-problems. This generates conflicting assignments than are repaired later with some heuristic. The main issue with this approach is that it relies heavily on the repair mechanism and that the problem decomposition stage consumes considerable computational time.

The third decomposition method is called Repeated Decomposition and Conflict Repair (RDCR). This approach seeks to decompose the problem in smaller sub-problems compared to GDCA and GDCR. This is achieved by tailored strategies to select tasks and workers for each sub-problem. Nine strategies for generating sub-problems were tested. This RDCR approach applies the decomposition followed by the MIP solver in an iterative way in order to repair conflicting assignments and obtain a valid overall solution. This results in RDCR being the fastest of the decomposition approaches while also producing best results.

This paper also presented experimental results comparing the proposed heuristic decomposition methods to solution generated in three other ways: optimal solutions (only for some small instances) by the MIP solver, solutions generated by a fast baseline heuristic and solutions generated by a human planner. Results of the comparison supported by a statistical analysis study allow to arrive to several conclusions as follows:

1. The proposed RDCR decomposition method is the best one overall when considering both solution quality and computational time.
2. The proposed GDCR decomposition method is slower than RDCR and appears to be the second best in terms of solution quality (although showing no significant difference to RDCR).
3. The GCDA decomposition method and the baseline heuristic show no significant difference in terms of solution quality, but the heuristic is the fastest method of all.
4. Compared to the reference solutions by the human planner, it is clear that any of the algorithms produces solutions of considerably better quality.

The research presented in this paper shows that although real-world home healthcare planning is a very difficult optimization problem and heuristics are often proposed in the literature to tackle this type of problems, applying modern MIP solvers within a decomposition strategy is an effective and efficient approach to tackle this problem.

Future work can be focused on the obvious aim of further improving the heuristic decomposition methods for better solution quality and shorter computational time. One particular direction to be explored is the implementation of the proposed heuristic decomposition methods using parallel computing as multiple sub-problems can be tackled simultaneously.

## Appendices

### Appendix 1: Statistical test on the RDCR experiments

In terms of solution quality, no statistical difference was found amongst the methods except with LBU-AF. However, the methods that showed statistical significant difference to LBU-AF were better, namely LBU-BF, RBK-BF, SBK-BF and SBK-WS. Table 5 reports results of this test with the calculated statistic on the left and the mean ranks on the right. The results show significantly difference between the nine methods with $\chi ^2(8) = 35.112, p<.001$. Therefore, we followed this with pairwise comparisons to identify differences between groups. The results showed significantly difference between the pairs LBU-AF:LBU-BF, LBU-AF:SBK-BF, LBU-AF:SBK-WS and RBK-BF:LBU-AF. It was concluded that LBU-AF produced lower solution quality (higher objective value). Then, we split the methods into two groups. One group with those methods that showed no statistical significant difference to LBU-AF, i.e. LBU-WS, SBK-AF, RBK-AF and RBK-WS. The other group with those methods that showed statistical significant difference to LBU-AF, i.e. SBK-WS, SBK-BF, RBK-BF and LBU-BF. The methods in the latter group achieved better solution quality on average.

**Table 5:** Friedman statistical test and mean ranks for the 9 decomposition rules in RDCR in respect of objective value

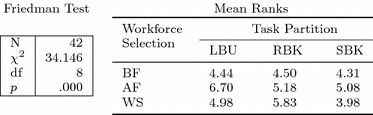

A lower mean rank indicates better better solution quality

In terms of computational time, the study identified three groups, with the methods giving lower computational time being LBU-BF and LBU-AF. Table 6 reports results of this test with the calculated statistic on the left and the mean ranks on the right. Statistical significant differences were found among the nine methods. Furthermore, Table 7 summarises the pairwise comparisons into three categories. The Positive column shows the number of other methods against which the method in the row spent more computational time with statistical significant difference. Similarly, the Negative column shows the number of other methods against which the method in the row spent less computational time with statistical significant difference. Then, the Indifferent column shows the shows the number of other methods against which the method did not reflect a significant difference on the computational time spent. As a result of this comparisons, we split the methods into three groups. In the first group are the faster methods: LBU-BF and LBU-AF. In the second group are those with mixed results hence in the middle of the ranking: RBK-BF, SBK-BF, RBK-AF, SBK-AF and LBU-WS. In the last group are the slower methods: RBK-WS and SBK-WS (Tables 8, 9).

**Table 6:** Friedman statistical test and mean ranks for the 9 decomposition rules in RDCR in respect of computational time

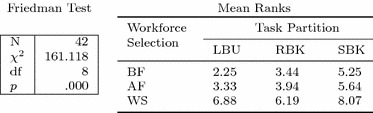

A lower mean rank indicates better (shorter) computational time

**Table 7:** Summation of differences in pairwise comparison between the 9 decomposition rules

Decomposition rule	Number of pairwise differences		
	Negative	Indifferent	Positive
LBU-BF	5	3	0
RBK-BF	3	4	1
SBK-BF	1	4	3
LBU-AF	5	3	0
RBK-AF	2	5	1
SBK-AF	1	4	3
LBU-WS	1	3	4
RBK-WS	0	4	4
SBK-WS	0	2	6

**Table 8:** Detailed results of the pairwise comparison between the 9 decomposition methods in RDCR, in respect of objective function value

Tested pair	Statistic	SE	Std. statistic	Sig.	Adj. sig.
IS-SBK:BF-LBU	.464	.598	.777	.437	1.000
IS-SBK:BF-SBK	.333	.598	.558	.577	1.000
IS-SBK:BF-RBK	.524	.598	.877	.381	1.000
IS-SBK:IS-LBU	1.000	.598	1.673	.094	1.000
IS-SBK:AF-SBK	1.107	.598	1.853	.064	1.000
IS-SBK:AF-RBK	1.202	.598	2.012	.044	1.000
IS-SBK:IS-RBK	1.857	.598	3.108	.002	.068
IS-SBK:AF-LBU	2.726	.598	4.562	.000	.000*
BF-LBU:BF-SBK	$-$.131	.598	$-$.219	.827	1.000
BF-LBU:BF-RBK	$-$.060	.598	$-$.100	.921	1.000
BF-LBU:IS-LBU	$-$.536	.598	$-$.896	.370	1.000
BF-LBU:AF-SBK	$-$.643	.598	$-$1.076	.282	1.000
BF-LBU:AF-RBK	$-$.7.38	.598	$-$1.235	.217	1.000
BF-LBU:IS-RBK	$-$1.393	.598	$-$2.331	.020	.712
BF-LBU:AF-LBU	$-$2.262	.598	$-$3.785	.000	.006*
BF-SBK:BF-RBK	.190	.598	.319	.750	1.000
BF-SBK:IS-LBU	.667	.598	1.116	.265	1.000
BF-SBK:AF-SBK	$-$.774	.598	$-$1.295	.195	1.000
BF-SBK:AF-RBK	.869	.598	$-$1.454	.149	1.000
BF-SBK:IS-RBK	1.524	.598	2.550	.011	.388
BF-SBK:AF-LBU	2.393	.598	4.004	.000	.002*
BF-RBK:IS-LBU	.476	.598	.797	.426	1.000
BF-RBK:AF-SBK	$-$.583	.598	$-$.976	.329	1.000
BF-RBK:AF-RBK	$-$.679	.598	$-$1.135	.256	1.000
BF-RBK-IS-RBK	$-$1.333	.598	$-$2.231	.026	.924
BF-RBK:AF-LBU	2.202	.598	3.685	.00	.008*
IS-LBU:AF-SBK	$-$.107	.598	$-$.179	.858	1.000
IS-LBU:AF-RBK	$-$.202	.598	$-$.339	.735	1.000
IS-LBU:IS-RBK	$-$.857	.598	$-$1.434	.151	1.000
IS-LBU:AF-LBU	1.726	.598	2.888	.004	.139
AF-SBK:AF-RBK	.095	.598	.159	.873	1.000
AF-SBK:IS-RBK	.750	.598	1.255	.209	1.000
AF-SBK:AF-LBU	1.619	.598	2.709	.007	.243
AF-RBK:IS-RBK	$-$.655	.598	$-$1.096	.273	1.000
AF-RBK:AF-LBU	1.524	.598	2.550	.011	.388
IS-RBK:AF-LBU	.869	.598	1.454	.146	1.000

* Statistically significant difference between pair at significant level 0.05

**Table 9:** Detailed results of the pairwise comparison between the 9 decomposition methods in RDCR, in respect of computational time

Tested Pair	Statistic	SE	Std. statistic	Sig.	Adj. sig.
BF-RBK:BF-LBU	1.190	.598	1.992	.046	1.000
BF-RBK:AF-RBK	$-$.500	.598	$-$.937	.403	1.000
BF-RBK:AF-LBU	.107	.598	.179	.858	1.000
BF-RBK:BF-SBK	$-$1.810	.598	$-$3.028	.002	.089
BF-RBK:AF-SBK	$-$2.202	.598	$-$3.685	.000	.008*
BF-RBK:IS-RBK	$-$2.750	.598	$-$4.602	.000	.000*
BF-RBK:IS-LBU	3.440	.598	5.757	.000	.000*
BF-RBK:IS-SBK	$-$4.631	.598	$-$7.749	.000	.000*
BF-LBU:AF-RBK	$-$1.690	.598	$-$2.829	.005	.168
BF-LBU:AF-LBU	$-$1.083	.598	$-$1.813	.070	1.000
BF-LBU:BF-SBK	$-$3.000	.598	$-$5.020	.000	.000*
BF-LBU:AF-SBK	$-$3.393	.598	$-$5.677	.000	.000*
BF-LBU:IS-RBK	$-$3.940	.598	$-$6.594	.000	.000*
BF-LBU:IS-LBU	$-$4.631	.598	$-$7.749	.000	.000*
BF-LBU:IS-SBK	$-$5.821	.598	$-$9.741	.000	.000*
AF-RBK:AF-LBU	.607	.598	1.016	.310	1.000
AF-RBK:BF-SBK	$-$1.310	.598	$-$2.191	.028	1.000
AF-RBK:AF-SBK	$-$1.702	.598	$-$2.849	.004	.158
AF-RBK:IS-RBK	$-$2.250	.598	$-$3.765	.000	.006*
AF-RBK:IS-LBU	2.940	.598	4.920	.000	.000*
AF-RBK:IS-SBK	$-$4.131	.598	$-$6.912	.000	.000*
AF-LBU:BF-SBK	$-$1.917	.598	$-$3.207	.001	.048*
AF-LBU:AF-SBK	$-$2.310	.598	$-$3.865	.000	.004*
AF-LBU:IS-RBK	$-$2.857	.598	$-$4.781	.000	.000*
AF-LBU:IS-LBU	$-$3.354	.598	$-$5.936	.000	.000*
AF-LBU:IS-SBK	$-$4.738	.598	$-$7.928	.000	.000*
BF-SBK:AF-SBK	$-$.3.93	.598	$-$.657	.511	1.000
BF-SBK:IS-RBK	.940	.598	1.574	.116	1.000
BF-SBK:IS-LBU	1.631	.598	2.729	.006	.229*
BF-SBK:IS-SBK	$-$2.821	.598	$-$4.721	.000	.000*
AF-SBK:IS-RBK	.548	.598	.916	.359	1.000
AF-SBK:IS-LBU	1.238	.598	2.072	.038	1.000
AF-SBK:IS-SBK	$-$2.429	.598	$-$4.064	.000	.002*
IS-RBK:IS-LBU	.69	.598	1.155	.248	1.000
IS-RBK:IS-SBK	$-$1.881	.598	$-$3.147	.002	.059
IS-LBU:IS-SBK	$-$1.190	.598	$-$1.992	.046	1.000

* Statistically significant difference between pair at significant level 0.05

### Appendix 2: Statistical test on the compariosn between decomposition methods

Table 10 shows the results of this analysis. On the left, results in terms of the objective function value (with $\chi ^2(4)=136.63, p<.001$) are shown for all five methods. On the right, results in terms of the computational time (with $\chi ^2(3)=111.71$, $p<.001$) are shown for all methods except the human planner as that solution was generated manually. With respect to the objective function value, results of the pairwise comparisons showed that almost all pairs of algorithm produced statistical significant different results. The exception were the pairs RDCR:GDCR and Heuristic:GDCA. With respect to the objective function value, results of the pairwise comparisons showed that there is statistical significant difference between the four methods, except between the pair GDCR:GDCA. Then, from this analysis, we conclude that in terms of objective function value, the better ranked methods are RDCR and GDCR and in terms of the computational time the better ranked methods are the heuristic and RDCR (Tables 11, 12).

**Table 10:** Friedman statistical test on objective value (left) and computational time (right) on five solution methods: GDCA, GDCR, RDCR, heuristic assignments and practitioner solution (objective value only)

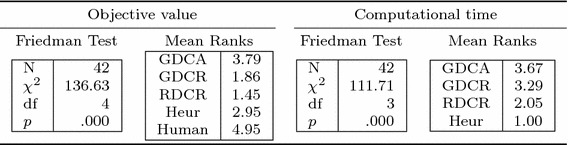

A lower rank presents a better approach

**Table 11:** Detailed results of the pairwise comparison between the five solution methods, in respect of objective function value

Tested Pair	Statistic	SE	Std. statistic	Sig.	Adj.sig.
RDCR:GDCR	.405	.345	1.173	.241	1.000
RDCR:Heuristic	1.500	.345	4.347	.000	.000*
RDCR:GDCA	2.333	.345	6.763	.000	.000*
RDCR:PT	3.500	.345	10.144	.000	.000*
GDCR:Heuristic	$-$1.095	.345	$-$3.174	.002	.015*
GDCR:GDCA	1.929	.345	5.590	.000	.000*
GDCR:PT	$-$3.095	.345	$-$8.971	.000	.000*
Heuristic:GDCA	.833	.345	2.415	.016	.157
Heuristic:PT	$-$2.000	.345	$-$5.797	.000	.000*
GDCA:PT	$-$1.167	.345	$-$3.381	.001	.007*

* Statistically significant difference between pair at significant level 0.05

**Table 12:** Detailed results of the pairwise comparison between the four automated solution methods, in respect of computational time

Tested Pair	Statistic	SE	Std. statistic	Sig.	Adj.sig.
Heuristic:RDCR	1.048	.282	3.719	.000	.001*
Heuristic:GDCR	2.286	.282	8.113	.000	.000*
Heuristic:GDCA	2.667	.282	9.466	.000	.000*
RDCR:GDCR	1.238	.282	4.395	.000	.000*
RDCR:GDCA	1.619	.282	5.747	.000	.000*
GDCR:GDCA	.381	.282	1.352	.176	1.000

* Statistically significant difference between pair at significant level 0.05
